# Design of the SHAPE-2 study: the effect of physical activity, in addition to weight loss, on biomarkers of postmenopausal breast cancer risk

**DOI:** 10.1186/1471-2407-13-395

**Published:** 2013-08-23

**Authors:** Willemijn AM van Gemert, Jolein I Iestra, Albertine J Schuit, Anne M May, Tim Takken, Wouter B Veldhuis, Job van der Palen, Harriët Wittink, Petra HM Peeters, Evelyn M Monninkhof

**Affiliations:** 1 Julius Center for Health Sciences and Primary Care, University Medical Center Utrecht, P.O. Box 85500 Hp, Str. 6.131, 3508 GA, Utrecht, The Netherlands; 2 Division of Public Health and Health Care, National Institute for Public Health and the Environment, P.O. Box 1 3720 BA, Bilthoven, The Netherlands; 3 Department of Health Sciences and EMGO Institute for Health and Care Research, VU University, Amsterdam, The Netherlands; 4 Shared Utrecht Pediatric Exercise Research (SUPER) Lab, Utrecht University, Utrecht, The Netherlands; 5 Child Development & Exercise Center, Wilhelmina Children’s Hospital, University Medical Center Utrecht, P.O. Box 85500 Hp, Str. 6.131, 3508 GA, Utrecht, The Netherlands; 6 Department of Radiology, University Medical Center, P.O. Box 85500 Hp, Str. 6.131, 3508 GA, Utrecht, The Netherlands; 7 Medisch Spectrum Twente Hospital, Department of Epidemiology, P.O. Box 50 000 7500 KA, Enschede, The Netherlands; 8 Department of Research Methodology, Measurement, and Data Analysis, University of Twente, Enschede, The Netherlands; 9 Research group Lifestyle and Health, Faculty of Health Care, Utrecht University of Applied Sciences, P.O. Box 85182 3508 AD, Utrecht, The Netherlands

## Abstract

**Background:**

Physical inactivity and overweight are two known risk factors for postmenopausal breast cancer. Former exercise intervention studies showed that physical activity influences sex hormone levels, known to be related to postmenopausal breast cancer, mainly when concordant loss of body weight was achieved. The question remains whether there is an additional beneficial effect of physical activity when weight loss is reached.

The aim of this study is to investigate the effect attributable to exercise on postmenopausal breast cancer risk biomarkers, when equivalent weight loss is achieved compared with diet-induced weight loss.

**Design:**

The SHAPE-2 study is a three-armed, multicentre trial. 243 sedentary, postmenopausal women who are overweight or obese (BMI 25–35 kg/m^2^) are enrolled. After a 4-6 week run-in period, wherein a baseline diet is prescribed, women are randomly allocated to (1) a diet group, (2) an exercise group or (3) a control group. The aim of both intervention groups is to lose an amount of 5–6 kg body weight in 10–14 weeks. The diet group follows an energy restricted diet and maintains the habitual physical activity level. The exercise group participates in a 16-week endurance and strength training programme of 4 hours per week. Furthermore, they are prescribed a moderate caloric restriction. The control group is asked to maintain body weight and continue the run-in baseline diet.

Measurements include blood sampling, questionnaires, anthropometrics (weight, height, waist and hip circumference), maximal cycle exercise test (VO_2peak_), DEXA-scan (body composition) and abdominal MRI (subcutaneous and visceral fat). Primary outcomes are serum levels of oestradiol, oestrone, testosterone and sex hormone binding globulin (SHBG).

**Discussion:**

This study will give insight in the potential attributable effect of physical activity on breast cancer risk biomarkers and whether this effect is mediated by changes in body composition, in postmenopausal women. Eventually this may lead to the design of specific lifestyle guidelines for prevention of breast cancer.

**Trial registration:**

The SHAPE-2 study is registered in the register of clinicaltrials.gov, Identifier: NCT01511276.

## Background

There is strong evidence that physical inactivity is associated with a higher postmenopausal breast cancer risk [1,2]. In contrast to most other risk factors, physical activity provides an opportunity for primary prevention.

The causal pathway through which exercise influences breast cancer risk is hypothesized to be predominantly hormone mediated, i.e. metabolic and sex hormones [3]. The evidence that oestrogens (endogenous as well as exogenous) contribute to breast cancer risk is strong and widely accepted [4-6]. Postmenopausal women with elevated levels of androgens also showed increased risk of developing breast cancer, even after adjustment for oestrogens [5-7]. In many cross-sectional observational studies, a low level of physical activity has been associated with higher serum concentrations of sex hormones in postmenopausal women [7-14], but not in all [15,16]. Physical activity might also influence postmenopausal sex hormones by increasing levels of sex hormone binding globulin (SHBG), resulting in lower amounts of unbound (free) active oestrogens and androgens in the circulation [10,11,14,17].

The beneficial effect of exercise on breast cancer related biomarkers might be partly explained by exercise-induced fat loss and prevention of becoming overweight or obese. Observational studies show associations between body mass index (BMI) and oestrogen levels in postmenopausal women [8-12]. Compared with normal-weight women, obese postmenopausal women have a higher blood concentration of oestrogens [12,13] and lower concentrations of SHBG resulting in increased levels of free oestradiol [12,14-16,18]. The association between BMI and androgens is less clear, i.e. cross-sectional studies reported conflicting results [10,19-21].

In our previous SHAPE trial we found that in the exercise group, reductions of sex hormone levels mainly occur when concordant loss of body fat was achieved [22]. These findings were in concordance with results from a comparable exercise intervention study [23,24]. Another exercise intervention study [25], however, found an overall intervention effect of exercise on sex hormones, which might be explained by the fact that in this study the overall difference in weight reduction between the intervention and control group was much greater compared with the earlier trials. A fourth trial investigating the effects of dietary, exercise and combined weight loss interventions, found that greater weight loss produced stronger effects on oestrogens and SHBG [26].

The question remains whether the beneficial effect of physical activity on breast cancer risk is fully explained by the accompanied weight loss, or whether physical activity has an additional positive effect on hormones.

Therefore, we set out to study the effect of weight loss mainly driven by exercise compared with equivalent weight loss driven by a diet only, on breast cancer risk biomarkers. Furthermore, we are specifically interested whether weight loss due to physical exercise induces greater amounts of fat loss (total and abdominal) and subsequently results in stronger favourable effects on relevant hormones compared with equivalent diet-induced weight loss.

## Methods/design

The aim of the SHAPE-2 study is to investigate the effect attributable to exercise on postmenopausal breast cancer risk biomarkers, when equivalent weight loss is achieved compared with diet-induced weight loss. The secondary aim is to study the effects of equivalent weight loss achieved by calorie reduced diet or by increased physical activity on body composition and fat distribution and whether this mediates sex hormone levels.

The SHAPE-2 study is designed as a three-armed, randomised controlled trial. The study programme runs in eight municipalities surrounding two research centres in the middle (Utrecht) and the east (Enschede) of the Netherlands. The total study duration for each study participant is about 21 weeks. After a 4–6 week run-in period, eligible women are randomly allocated to (1) a diet group; (2) an exercise group or (3) a waiting list control group. Both intervention groups have the aim to lose an amount of 5–6 kg of bodyweight in 10–14 weeks. The intervention period is followed by a weight maintenance period lasting at least 2 weeks.

The study protocol is approved by the Medical Ethics Committee of University Medical Centre Utrecht, in accordance with the Helsinki declaration, before the start of data collection.

### Study population

A total of 250 postmenopausal women, aged 50–69 years, are included. Eligible women are overweight or obese (BMI 25–35 m/kg^2^), have a sedentary lifestyle and live in the middle or east of the Netherlands.

Postmenopausal state is defined as natural cessation of menses for at least 12 months, or in case of hysterectomy: aged 55+ and likely to be postmenopausal based on medical history. Sedentary is defined as less than 2 hours of moderate-to-vigorous physical activity per week (≥4 metabolic equivalents (MET)) [27]. Energy expenditure from occupational activity (except for highly active jobs e.g. courier, sports instructor), walking at moderate pace and cycling as a transport medium (<16 km/hour) are not considered. In case of doubt, individuals are discussed in the study team. Exclusion criteria are factors that either interfere with endogenous sex hormone levels or successful completion of the diet or exercise intervention (see Table 1).

**Table 1:** SHAPE-2 study inclusion and exclusion criteria

**Inclusion criteria**	**Exclusion criteria**
Female	Presently using sex hormones
50-69 years of age	Use of beta-blockers or oral corticosteroids
Postmenopausal (last menses >12 months)	Smoking
Body mass index (BMI) 25–35 m/kg^2^	Alcohol or drug abuse
Sedentary lifestyle (<2 hours/week of at least moderately intensive activities (≥4 MET))	Diagnosed with breast cancer (present or history)
Willing to be randomly assigned to one of the three study arms	Diagnosed with other cancer (present or <5 years of history), except fornon-melanoma skin cancer
Informed consent for all screening and study activities	Diabetes mellitus or other (unstable) endocrine related diseases
	Any disorder that might impede participation in the exercise programme
	Following, or intention to follow, a structured weight loss programme elsewhere
	Investigators opinion (successful fulfilling of the programme is highly unlikely)

### Recruitment and screening

**Figure 1:**
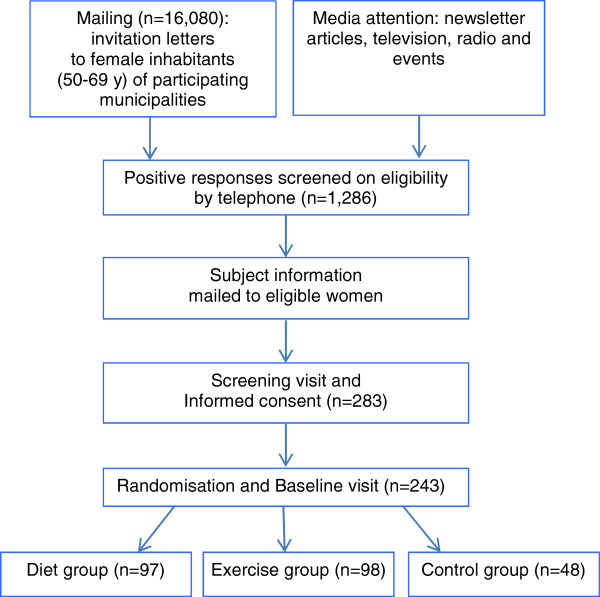
Flow-chart recruitment and inclusion procedure.

Study participants are mainly recruited by invitation letters explaining the study goals and inclusion criteria. These letters are sent to a random selection of female inhabitants (aged 50–69 years, Dutch nationality) of the participating municipalities: Zeist, Bilthoven, Utrecht, Nieuwegein, Houten, IJsselstein, Enschede, and Oldenzaal. Furthermore, we aim to publish articles in local newspapers including calls for participants. Responding women are contacted by phone by a study nurse to provide more information and to further assess eligibility. Potential candidates are invited for a screening visit at the research unit in their region, where informed consent is signed and BMI and glucose (ACCU-CHEK® Aviva) are checked. Additionally, motivation and physical ability to perform the exercise programme are discussed. If eligible, the participant is scheduled for the study dietitian and starts with the run-in period. See Figure 1 for the flow chart of the recruitment and inclusion procedure. All participating women gave informed consent before start of the study.

### Baseline diet during run in period

During the 4–6 week run-in period, a baseline diet is prescribed which resembles the habitual intake of the participant and is in accordance with the Dutch Guidelines for a Healthy Diet [28] (50-60% carbohydrate, 15-20% protein and 20-35% fat). The energy content of the baseline diet is determined using the individual’s habitual energy intake (dietary history), body weight history and a calculated estimate using the Harris & Benedict formula [29] multiplied by an estimate of their Physical Activity Level (PAL). Special attention is paid to alcohol intake (maximum of one drink per day) and fibre intake (minimum of 25 grams per day) since these may influence sex hormone levels [30-32]. The run in period and the baseline diet aim to normalise the dietary pattern, stabilise body weight, check the estimated energy requirements and evaluate protocol adherence.

During the run-in, adherence to the programme is monitored by filling out a 3-day food diary, weekly self-weighing and telephone contacts with the dietitian (see Table 2).

**Table 2:** Overview study programme, contact moments and measurements

	**Run-in phase**						**Intervention phase**														**Maintenance**	
week	−6	−5	−4	−3	−2	−1	1	2	3	4	5	6	7	8	9	10	11	12	13	14	15	16
**Diet group**																						
-diet composition	Baseline diet						Energy restricted diet (−500 kcal/day)															
-dietary counselling*	F		T		T		F	G	T	G	T	G	T		T	G	T			F, G		T
**Exercise group**																						
-diet composition	Baseline diet						Energy restricted diet (−250 kcal/day)															
-dietary counselling*	F		T		T		F		T		T		T		T		T			F		T
-exercise programme**							Aerobic and strength training (4 hours/week)															
							E	E	E	E	E	E	E	E	E	E	E	E	E	E	E	E
**Control group**																						
-diet composition	Baseline diet						Baseline diet															
-dietary counselling*	F		T		T		T				T											T
Measurements***																						
-Habitual dietary intake (dietary history)	X																					
-Actual dietary intake (3-day food records)				X						X											X	
-Habitual physical activity (questionnaires)							X															X
-Actual physical activity (accelerometer)	X																				X	
-Anthropometrics (weight, waist, hip circumference); body composition (DEXA); visceral and subcutaneous abdominal fat (MRI); fitness (maximal exercise test); blood pressure; sex hormones (blood sampling)							X															X

*dietary counselling: F = face-to-face individual counselling; T = individual counselling by telephone; G = group counselling session.

** exercise programme: E = group fitness and Nordic walking, 4 hours per week.

*** measurements: X = all participants.

### Randomisation & intervention

After successful completion of the run-in period, subjects are randomised to (1) a diet group; (2) an exercise group or (3) a waiting list control group. Randomisation is performed via a computer-generated sequence, stratified per municipality, in block sizes of 5 (ratio interventions vs. control; 2:2:1).

The goal of both intervention programmes is to lose an equivalent amount of 5–6 kg of body weight, in 10–14 weeks’ time. The weight loss interventions are supervised by dietitians and physiotherapists, established in each participating municipality.

Body weight is closely monitored in both intervention groups by continuation of weekly self-weighing. Supervised weighing, by the dietitian (at every visit) and physiotherapist (weekly), is performed in addition. Participants, whose weekly weight loss do not meet or exceed the 0.5 kg/week loss for 3 consecutive weeks, receive extra coaching to adapt their diet or exercise level.

If the weight loss goal is reached, or after a maximum of 14 weeks, a weight maintenance period (2–6 weeks) starts in which energy intake and energy expenditure is balanced by dietary adaptations. The goal of this maintenance period is to establish stable weight in order to obtain stable levels of sex hormones.

#### Diet group: weight loss induced by diet only

The weight loss intervention is delivered by registered dietitians, experienced in treatment of overweight and motivational interviewing. Motivational interviewing is a client-centred counselling approach which is a proven effective method used to increase motivation and to establish behaviour change [33,34].

Frequent contacts with the dietitian are scheduled (see Table 2 for an overview of the study programme). After randomisation, women individually meet their dietitian for the prescription of a calorie restricted diet. The diet has a deficit of 500 kcal/day as compared with the individuals energy requirements estimated at the run-in period. The diet is composed of the same proportions of macronutrients as the baseline diet conform National Guidelines for a Healthy Diet [28]. Additionally, 5 one-hour interactive group sessions are planned (maximal 12 women/group). The programme for these sessions is based on principles of cognitive behavioural therapy [35] and motivational interviewing [34] and consists of nutrition education, behaviour change techniques and self-management training. Adherence to the programme is monitored by completing a 3-day food diary and frequent telephone contacts with the dietitian (see Table 2). Women in the diet group are requested to maintain their habitual physical activity level.

#### Exercise group: weight loss mainly induced by exercise

Women randomised to the exercise group are enrolled in a 16-week exercise training programme, delivered by physiotherapists. Additionally, a moderate caloric restriction of 250 kcal/day is prescribed by a dietitian in an individual session. From recent literature, we know that achieving and maintaining a body weight reduction by exercising in untrained and obese women is a long term process and a goal hard to attain [36,37]. Compensatory mechanisms both physically and mentally, and behavioural reasons withhold the person from losing weight adequately [38,39]. We, therefore, decided to study the effect of exercise in combination with a slight diet energy deficit.

The prescribed diet is monitored by regular telephone contacts with the dietitian. In this group, main emphasis is placed on the exercise programme, which contains four hours of moderate-to-vigorous exercise per week in group- and individual sessions. The estimated energy expenditure of the exercise programme is approximately 350 kcal/day, based on corresponding MET rates [27]. For our specific study population, we corrected METs for age [40,41].

The exercise protocol contains both endurance and strength training. Levels of exercise intensity are gradually increased during the study programme. Intensity of strength training is determined by pragmatic 20- and 15-repetition maximum (RM) tests, performed several times throughout the 16 weeks to adapt the resistance.

Intensity of endurance training is determined by the heart rate reserve based on the guidelines of the American College of Sports Medicine, adapted for older women [42]. Target heart rates are based on results of a maximal exercise test and calculated by the formula: [intensity%*(maximal heart rate – resting heart rate)] + resting heart rate. Study participants wear heart rate monitors while exercising and receive a badge with individually calculated target heart rate zones for a range of training intensities.

During every training session, subjects fill in an exercise log which is used as a tool to adhere to the protocol and for monitoring by the physiotherapist.

#### Group exercise

Twice a week, subjects participate in a standardised one-hour group session, facilitated by a physiotherapist. Groups consist of 5–6 women. The group exercise sessions include 20–25 minutes high-intensity endurance training combined with 25 minutes strength training. Classes start and end with a 5–10 minute warming up and cooling down, respectively.

Endurance training is performed in circuits on several exercise machines, e.g. a treadmill, cycle or cross-trainer. Intensity is gradually increased (see Table 3).

**Table 3:** Group fitness training programme

**Week**	**Endurance**	**Strength**
**1-3**	40-60% HRR*	1 circuit of 20–25 repetitions. Weights based on 20-RM^#^
		Exercises: legs (squat, lunges, calve raises), arms (biceps curl, triceps extension), shoulder (shoulder press), thorax (Barbell bench press), back (rowing). Abdomen: crunch 30–40 repetitions.
**4-8**	60-70% HRR* 15–20 min,	
	Plus 70-89% HRR* 5–10 min	
**9-12**	Interval training: 10 × 30 sec. vigorous to maximal exercise, alternated with 1 min active rest	2 circuits of 15–20 repetitions. Weights based on 15-RM^#^
		Exercises: legs (squat), arms (biceps curl, triceps extension), shoulder (shoulder press), thorax (Barbell bench press), back (rowing). Abdomen; crunch 30–40 repetitions; hoover 2× 45 seconds.
	Plus 10 min 60-75% HRR* endurance	
**13-16**	Interval training: 2 circuits of 8 × 30 sec vigorous to maximal, alternated with 1 min active rest	
		If in time calve raises and lunges can be added.
	Plus 5 min 60-75% HRR* endurance	

*HRR: Heart rate reserve.

#RM: Repetition maximum.

The standardised strength training protocol includes exercises for the major muscle groups which is also performed in circuits (Table 3).

#### Individual exercise

For feasibility reasons, individual home-based exercise is also included. It comprises two hours of Nordic walking at 60-65% of the heart rate reserve. All participants receive Nordic walking poles and instructions. If due to medical reasons Nordic walking is not desirable, a proper alternative is sought, e.g. swimming laps or cycling (vigorous effort). Supervised lessons of Nordic walking by instructors are organised to increase motivation and compliance. These sessions can be attended voluntarily, however, women are strongly encouraged to join.

Home-based Nordic walking sessions are registered in an exercise-log, which are checked regularly by the physiotherapist. Group sessions at the physiotherapists office and Nordic walking lessons are regularly monitored by the researchers.

#### Control group: stable weight

Participants in the control group are requested to keep their weight stable by adhering to the baseline diet, and maintaining their habitual exercise pattern. They are offered an alternative weight loss programme after the study period, consisting of 4 dietary group sessions and several exercise classes such as Nordic walking and/or fitness.

### Outcomes and measurements

Study participants visit the research centre for measurements twice: at baseline (i.e. the end of the run-in period) and at the end of the study (see Table 2). Measurements include blood sampling, anthropometrics: height (at baseline), weight, waist- and hip circumference), a total body DEXA scan, abdominal MRI, blood pressure and cardiorespiratory fitness. At every visit, information on medication use is assessed. Furthermore, we assess information on socio-demographic variables, general health, medical history, reproductive history and smoking history at baseline by a self-constructed questionnaire.

#### Blood samples

Blood samples (30 ml) are drawn in order to determine serum concentrations of oestradiol (total and free), oestrone, testosterone and SHBG. After centrifugation, samples are directly stored at −20°C and at −80°C within one week. All samples from one individual will be analysed in the same batch since the batch-to-batch variation can be higher than any woman’s likely change in hormones over the year [43]. Serum oestrogens and testosterone will be determined by use of the LC-MS method, in the UHSM, Manchester laboratory [44]. SHBG will be measured by commercially available double-antibody radioimmunoassay kits (Roche Cobas: SHBG-03052001), performed in the laboratory ”Stichting Huisartsenlaboratorium Oost” in Velp [45]. Technicians are blinded to study allocation.

#### Anthropometrics and body composition

Body weight and height are measured while the subjects wear light clothes without shoes. To measure body weight, we use calibrated analogue balance and digital balance scales (SECA®), depending on study centre. Subjects are always measured on the same balance scale. Analogue values are rounded to the nearest 0.5 kg. Height is measured using a wall mounted tape measure and rounded to the nearest 0.5 cm.

Waist circumference (to the nearest 0.5 cm) is measured standing at the midway between lower ribs and iliac crest. Hip circumference (to the nearest 0.5 cm) is measured standing over the buttocks. All measurements are taken in duplicate and averaged.

Total body fat (kg) and fat percentage (%) are assessed by a total body DEXA-scan (Lunar, Prodigy™). The DEXA scan measures body composition according to a three-compartment model: fat mass, lean tissue, and bone mineral content. The standard soft tissue analysis is performed using software supplied by the manufacturer.

Visceral abdominal fat (VAT) and subcutaneous abdominal fat (SAT) are measured by MRI (Philips, Ingenia 1.5 T) with the use of the three-point IDEAL method, described by Dixon [46].

#### Blood pressure

Blood pressure is measured by an automatic tonometer (OMRON M4 +) after a minimum of 5 minutes rest. Measurements are taken twice, with a 2-minute time interval.

#### Cardiorespiratory fitness

A maximal cycle exercise test with respiratory gas analysis is performed to measure cardiorespiratory fitness, defined by the highest oxygen uptake during the test (VO_2peak_).

All testing is conducted according to the ATS guidelines [47]. Subjects are tested on a bicycle ergometer (Ergoline, type Ergoselect 200 P, CareFusion, Houten, the Netherlands and Jaeger ER800®, Würtzburg, Germany). Seat height is adjusted so that subject’s legs are near full extension during each pedal revolution.

The ramp cycle test protocol starts with 2 minutes of rest and 3 minutes of active rest (cycling without workload). The test phase consists of 24-second stages of graded exercise. Workload increases with 12.5 Watt or 15 Watt at every step, depending on the predicted maximum Watt per subject. Pedalling speed is kept around 65 revolutions per minute (RPM). If participants fail to keep up or drop below 40 RPM, the test is ended and followed by a 2-minute recovery phase. The maximal exercise test is performed under medical supervision. During the test, a 12-lead electrocardiogram (ECG) and respiratory data through breath-by-breath analysis (Oxycon Pro®, Jaeger, by Care Fusion, Houten, The Netherlands) are continuously measured. Heart rate is determined from the ECG. Cuff blood pressure is monitored before and throughout the exercise and recovery phase. VO_2peak_ is defined as the highest 15-second average of VO_2_ obtained at the end of the test and is expressed as ml/min and ml/kg/min.

The goal of the maximal cycle exercise test is threefold. In addition to measuring cardiorespiratory fitness/VO_2peak_, it also serves as a medical evaluation, and maximal heart rate is used to estimate training intensity for women participating in the exercise programme.

#### Physical activity

Physical activity level is assessed with an activity monitor (GT3X + Tri-Axis Actigraphy Monitor, ActiGraph®). This non-invasive device provides information on individual activity including energy expenditure, sedentary behaviour, activity intensity levels, and METs. It is worn in 7 consecutive days during the run-in and maintenance period.

Furthermore, validated physical activity questionnaires are used to measure habitual activity level (PASE questionnaire) [48] and short-term physical activity level (SQUASH questionnaire) [49] at baseline and at the end of study.

#### Dietary intake

Habitual dietary intake is assessed at baseline using the dietary history method. Actual dietary intake and adherence to the diet plan are assessed using 3-day food records (including 1 weekend day) during every study period (run-in, intervention and maintenance, see Table 2). Participants are instructed by the dietitian how to complete the records. Filled-in records are checked by the dietitian for completeness and can be discussed with participants in the next telephone contact. Energy intake and nutrient composition are calculated using the Dutch Food Composition Database [50].

### Sample size

Sample size calculations are based on the effect of the interventions on the primary outcome, i.e. serum oestradiol levels. The following comparisons will be made:

1) diet-induced weight loss versus exercise-induced weight loss. 2) diet-induced weight loss versus control. 3) exercise-induced weight loss versus control. First, we calculated the sample size for the first comparison, i.e. diet-induced weight loss (n = 85) versus exercise-induced weight loss (n = 85), since the difference in oestradiol levels between these groups is expected to be the smallest (8%). Based on the estimated sample sizes, we calculated the number of subjects needed in the control group (n = 36). The sample size of the control group can be much smaller, since the expected difference with the interventions groups is large (12% and 20%, respectively). The sample size calculations resulted in the following estimated numbers per group, taking into account 5% drop out and 15% non-compliance: control group n = 45, diet group n = 104 and exercise group n = 104.

### Statistical analysis

Descriptive statistics will be used to characterise the study population at baseline per study arm. Baseline and end of study values of sex hormone levels, total body fat and intra-abdominal body fat will be tabulated by treatment group. Sex hormones will be log transformed and geometric means will be presented if not normally distributed.

The main analysis will be performed according to the intention-to-treat principle, by linear regression analysis, where outcomes for patients are analysed by assigned treatment, regardless of the level of adherence. As a secondary analysis, adherence will be examined as a potential modifier of the intervention effects.

A per-protocol analysis will be performed analysing women who reached the weight loss goal only. Whether changes in body fat (total, abdominal) mediate or moderate intervention effects on sex hormone levels will be explored.

## Discussion

In the SHAPE-2 study, we aim to investigate the potential effect of physical activity on biomarkers of breast cancer risk (sex hormones), additional to weight loss. We hypothesize that exercise-induced weight loss results in a stronger decrease in serum sex hormones compared with equivalent diet-induced weight loss and compared with controls.

The goal of the intervention, and challenge in this trial, is for subjects to lose an equivalent amount of 5–6 kg of body weight in both intervention groups. Success of the study depends heavily on subjects’ adherence and motivation.

We implemented several strategies to increase adherence and motivation of the study subjects. First, the weight loss interventions are delivered by experienced dietitians and physiotherapists, who are situated in the municipality and easily accessible. For participants in the intervention groups, we scheduled a high contact frequency with the dietitian and physiotherapists which is proven to be a relevant success factor for weight reduction [51]. Second, group sessions are implemented in both intervention programmes which provide a combination of social support, a healthy dose of competition and increase self-efficacy [52]. It is also suggested that dietary group sessions produce greater weight loss effects than individual counselling alone [53]. Groups are kept small to secure enough room for interactions and tailoring of the programme to the specific needs of the participants. Third, to strengthen motivation of the participants and for monitoring purposes, the researchers visit diet and exercise group sessions regularly. Furthermore, participants receive newsletters about the study or related topics and the study website is updated frequently.

Adherence of the control group is also a challenge. We expect women allocated to the control group to be disappointed since they have their mind set on losing weight. This might lead to a change in lifestyle, either conscious or unconscious, resulting in slight weight loss. To anticipate, we repeatedly stress the importance of the control group. Moreover, we offer an evenly attractive alternative weight loss programme starting at the end of the study.

This trial has a strict time schedule. We aim to include 25–30 participants per municipality in a small time window, since the participants have to start simultaneously with the group interventions. The first group starts at the central research site in the Utrecht region. Inclusion in the second participating region (Enschede) starts in parallel. Consecutive groups within one region start after a minimum time interval of 1 month.

Due to a consecutive inclusion of municipalities, the winter season, summer holidays and national holidays (e.g. Christmas) might affect the adherence of some study groups. To retain compliance in order to achieve the same weight loss goal, dietitians and physiotherapists will anticipate these circumstances. We pay extra attention to the Nordic walking programme in winter since the colder climate and shorter days may threat compliance. We therefore provide more options for supervised Nordic walking hours and reflective clothing in winter.

In our study, we will measure visceral and subcutaneous abdominal fat by using MRI, which is a highly sensitive technique to measure changes in (intra-)abdominal fat. A side effect of the use of cross sectional imaging in healthy subjects is that there is a risk of incidental findings. Rates of over 30% have been described while the proportion of subjects that might benefit from these findings is likely to be much lower than the proportion that will have no or even an adverse effect [54,55]. Outweighing the risk-benefit ratio and ethical considerations [56], we decided on the following procedure. The non-contrast enhanced T1-weighted IDEAL scans are considered non-diagnostic and therefore will not be routinely reviewed by a radiologist. If researchers encounter an apparent finding that strikes them as a possible abnormality, the radiologist will be consulted. When the finding is of potential clinical relevance, the participant and their general practitioner will be informed and advised on further work up.

Our trial is the first study especially designed to assess the additional effect of exercise on sex hormone levels when equivalent weight loss is achieved. Four former exercise intervention studies in the field suggested an interplay between sex hormones and body weight/fat mass [22-26]. Greater weight loss produced greater effects on serum sex hormone levels and SHBG.

The question remains whether there is an additional effect of exercise on serum sex hormones, as breast cancer risk biomarkers, when equivalent weight loss is reached. In the SHAPE-2 study, we aim to investigate the potential effect attributable to physical activity on postmenopausal breast cancer risk biomarkers, in addition to weight loss.

## Competing interests

This work is supported by the Dutch Cancer Society [UU 2010–4843]. The support from the sponsor is unconditional, and the data collection, design, management, analysis, interpretation and reporting will be performed without their interference. We have no non-financial competing interests to disclose.

## Authors’ contributions

Authors WG, JI, PP, AS, AM, TT, JP and EM were involved in the study design. TT, advised in the composition of the exercise programme. JI, EM and WG composed the dietary programme. WV designed the MRI scan protocol. HW supplied services and materials for exercise testing. WG, EM, PP and JS participated in the management and coordination of the study. JP participated in the coordination on the second research site. WG and EM drafted the manuscript. All have been involved in revising the content of the manuscript. All authors have read and approved the final manuscript.

## Authors’ information

Willemijn van Gemert, MD, works as a PhD candidate at the department of epidemiology of the Julius Center. Jolein Iestra, PhD, is a dietitian at the department of Public Health of the Julius Center. Petra Peeters, MD, PhD, Evelyn Monninkhof, PhD, and Anne May, PhD are epidemiologists working at the epidemiology department of the Julius Center. Albertine Schuit, PhD, is an epidemiologist at the national institute of Health and VU university. Tim Takken, PhD, is an exercise physiologist working at the UMC Utrecht. Wouter B. Veldhuis, MD, PhD, is a radiologist working at the radiology department of the UMC Utrecht. Job van der Palen, PhD, is an epidemiologist and works as a scientific research coordinator in the Medical Spectrum Twente and as a professor in evaluation and assessment in healthcare research at the University Twente. Harriët Wittink, PhD, is a physiotherapist and professor Lifestyle and Health at the faculty of health care, Utrecht University of Applied Sciences.

## Pre-publication history

The pre-publication history for this paper can be accessed here:

http://www.biomedcentral.com/1471-2407/13/395/prepub
